# 12 Weeks of Supervised Team Sports in Danish Municipal Health Center Lowers Systolic Blood Pressure and Increases Performance in Hypertensive Chronic Obstructive Pulmonary Disease and Type 2 Diabetes Mellitus Patients

**DOI:** 10.3390/jfmk10020209

**Published:** 2025-06-04

**Authors:** Jesper Atti, Julie Kissow, Jens Bangsbo

**Affiliations:** The August Krogh Section for Human and Molecular Physiology, Department of Nutrition, Exercise and Sports (NEXS), University of Copenhagen, DK-2100 Copenhagen, Denmark; jpa@nexs.ku.dk (J.A.); jkk@nexs.ku.dk (J.K.)

**Keywords:** hypertension, team sports, chronic disease management

## Abstract

**Background:** Hypertension is a significant public health issue, particularly in individuals with comorbidities such as COPD and T2DM, which exacerbate cardiovascular risk and impair quality of life. While physical activity is an effective intervention for reducing blood pressure and improving health markers, conventional therapies often lack the social and psychological benefits of team sports. Team sports conducted as small-sided games provide a dynamic, engaging approach that combines physical, social, and psychological advantages, making them particularly suitable for individuals with complex chronic conditions. **Methods:** This non-randomized intervention study involved twenty-eight hypertensive patients, including 16 individuals with type 2 diabetes mellitus (T2DM) (8 men and 8 women) and 12 with chronic obstructive pulmonary disease (COPD) (7 men and 5 women). Participants engaged in a training program, primarily consisting of team sports (floorball and cone ball), at a municipal health center twice a week for 12 weeks. **Results:** The intervention led to a significant reduction in systolic blood pressure (*p* = 0.006), with patients with COPD and T2DM showing decreases of 9.6 ± 12.7 mmHg and 12.4 ± 19.0 mmHg, respectively. Additionally, the time to complete the 2.45 m “Up and Go” test improved significantly (*p* < 0.001), with both COPD (*p* = 0.011) and T2DM (*p* = 0.005) patients demonstrating notable improvements. However, no significant changes were observed in body mass, chair stand performance, five-repetition sit-to-stand test, handgrip strength, or diastolic blood pressure following the intervention. **Conclusions:** Team sports training conducted in a municipality health center is effective in lowering blood pressure and improving functional capacity in hypertensive COPD and T2DM patients.

## 1. Introduction

Hypertension is a major public health concern, particularly among individuals with chronic comorbidities such as obstructive pulmonary disease (COPD) and diabetes (T2DM). These conditions not only worsen the prognosis of hypertension but also increase the risk of cardiovascular events and lead to diminished quality of life [1,2,3]. The management of hypertension in patients with these comorbidities is challenging, requiring a multifaceted approach to control blood pressure and mitigate associated risks [4].

Physical activity is widely recognized as an effective intervention for reducing blood pressure, improving cardiovascular function, and enhancing metabolic health. Both COPD and T2DM patients are associated with diminished cardiovascular and muscular fitness, which collectively impair functional capacity and increase cardiovascular risk [5,6]. Several studies have demonstrated the positive impact of exercise on hypertensive patients; however, research has predominantly focused on controlled clinical trials [7,8]. These controlled environments, while valuable, may not fully capture the complexity of real-world behaviors and adherence to exercise regimens, particularly in patients with COPD and T2DM.

Team sports conducted as small-sided games represent a dynamic and engaging form of physical activity that has been shown to offer physiological benefits such as reduced LDL cholesterol, lower body and visceral fat content, reduced blood pressure and mean arterial pressure (MAP), and increased functional capacity [9,10]. Team sports may also offer additional benefits beyond those seen with individual exercise programs [11,12]. The social and competitive elements of team sports can improve motivation, adherence, and psychological well-being, which are often compromised in individuals with chronic conditions [13]. Furthermore, the communal aspect of team sports may enhance cardiovascular and metabolic outcomes through more consistent participation and enjoyment [14]. And, while conventional exercise therapies can be beneficial for patients with T2DM and COPD, conventional exercise therapies may only focus on single modalities, such as aerobic or resistance training, and lack social interaction [15,16].

Hereby, team sports are suitable for patients with T2DM and COPD due to the combination of physical, social, and psychological benefits.

Thus, the aim of the present study was to examine the physiological adaptations and functional changes in hypertensive COPD and T2DM patients when participating in regular, mainly team sports training in a municipality health center, where we expected to see a reduction in blood pressure, mainly systolic, due to the intermittent structure in team sports. By conducting the study in a real-world setting, we hope to provide a more comprehensive understanding of how team sports can affect blood pressure, improve health markers, and enhance the quality of life in this high-risk population. The insights gained will contribute to the development of practical and sustainable interventions for managing hypertension in patients with complex chronic diseases.

## 2. Methods

### 2.1. Subjects

Thirty-five hypertensive participants were screened and included in the project. Due to absence at the post-test, a total of 28 hypertensive participants completed the intervention. Participants were aged 66.5 ± 9.2 (mean ± SD; range: 47–79) years and with a body mass of 94.4 ± 24.8 kg with either chronic obstructive pulmonary disease (COPD; n = 16; eight men and eight women) or type 2 diabetes mellitus (T2DM; n = 12; seven men and five women) took part in training at a municipal health center (Table 1). The inclusion criteria required participants to be referred by their primary physician. The physio at the municipal health center then deemed them safe for participating in the intervention after an interview. Exclusion criteria specified that individuals who had engaged in regular physical activity (at least two sessions per week) within the last 12 months prior to the intervention were not eligible for participation. Participants meeting these criteria were deemed eligible to enroll in the study.

The study received approval from the Committee on Health Research Ethics, Region of Copenhagen (H-23000075), and was conducted in accordance with the principles outlined in the Declaration of Helsinki. All participants were informed about potential risks and discomforts related to the experiments. The study was a cooperation between the Copenhagen Center for Team Sports and Health, the University of Copenhagen, and the municipality of Brøndby, Denmark.

### 2.2. Training

The training intervention lasted 12 weeks, and training was scheduled twice a week. Every training session had a duration of 60 min and was initiated with a 10 min warm-up period. The training was carried out in groups of 6–8 participants according to their condition, with the COPD patients training together and the T2DM patients with each other. The training consisted of team sports, such as floorball or cone ball, where floorball is a game played with plastic hockey sticks. A point is scored when the ball is shot into the goal. Only goals scored using the stick are counted. Cone ball is a game where a point is scored when a player throws the ball at a cone, knocking it over. The player who knocks over the cone must then take it back to their own baseline and place it among their team’s cones. Both ball games were played as small-sided games (3v3–4v4) in 6–8 min separated by 3–4 min rest periods or strength training conducted as circuit training with 2–3 sets of 10–15 repetitions for each exercise. The training schedule is presented in Table 2, showing that 75% of the training sessions were team sports. COPD and T2DM patients completed 80–100% of all training sessions. All missed training sessions were due to illness or personal circumstances.

As a note, team sports offer the flexibility to modify participation levels to accommodate individuals with varying physical abilities. For instance, participants with initial lower-limb weakness can engage in the activity from a seated position, enabling them to participate while minimizing physical strain. This adaptive approach promotes inclusivity and provides gradual progression to standing participation as their strength and functional capacity improve.

### 2.3. Measuring and Test Procedures

The participants conducted different measurements and tests individually at the municipal health center. All tests were conducted by staff from the department of Nutrition, Exercise and Sports (NEXS) at the University of Copenhagen.

#### 2.3.1. Physiological Measures

Blood pressure was measured at least three times (average n = 3) in a relaxed, seated position on the upper arm before training in the afternoon, using an automatic blood pressure monitor (Omron M3 Comfort) after participants had rested for 3–4 min. If the third reading differed by more than 10 mmHg for systolic or 5 mmHg for diastolic blood pressure compared to the second reading, a fourth measurement was taken, and the average of all measurements was calculated. Following blood pressure measurements, body mass was assessed using a standardized body scale (Tanita BC 587).

#### 2.3.2. Performance Tests

On a separate day, five standardized performance tests were conducted to assess functional capacity [12]: (1) participants performed as many sit-to-stand repetitions as possible within 30 s from a 45 cm armless chair; (2) time was measured for a 2.45 m “Up and Go” test, starting from a seated position, walking 2.45 m and then around a cone, and returning to sit; (3) maximal 30 s biceps curls using weights of 2.23 kg for women and 4.46 kg for men; (4) maximal hand-grip strength with a hydraulic dynamometer; and (5) total distance covered during a 6 min walk test between two cones set 20 m apart. Participants received guidance and encouragement throughout, with time monitored and resting permitted during the walk test as needed.

### 2.4. Statistics

All statistical analyses were performed using IBM SPSS Statistics (version 24; IBM, Armonk, NY, USA). Data distribution was assessed for normality using Q-Q plots and the Shapiro–Wilk test. Within-group effects were analyzed using paired *t*-tests to evaluate changes from pre- to post-intervention and to test the hypothesis of a significant effect over 12 weeks. Data are presented as mean ± standard deviation (SD). A significance level of *p* ≤ 0.05 was considered statistically significant.

## 3. Results

### 3.1. Blood Pressure

**Figure 1:**
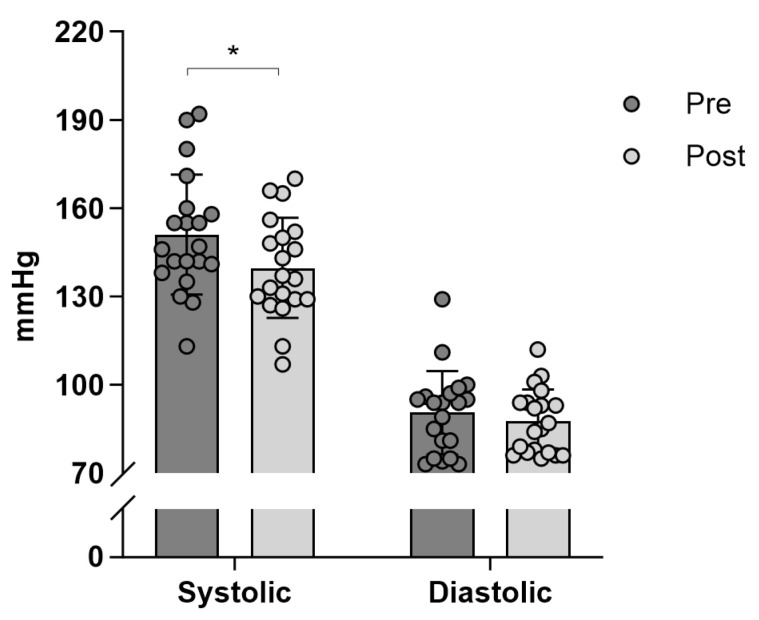
Systolic and diastolic blood pressure (mmHg) within a group of obstructive pulmonary disease (COPD) and diabetes mellitus type 2 (T2DM) patients before (Pre) and after (Post) 12 weeks of team sports and circuit training in a municipal health center. Data are presented as individual values and mean ± SD. n = 8 for COPD and n = 12 for T2DM. * Denotes difference (*p* < 0.05) between Pre and Post.

After the training intervention systolic blood pressure was 7.5% lower (*p* = 0.006) compared to before the intervention corresponding to a decrease of 11.3 mmHg (95% CI −19.0 to −3.6 mmHg, Figure 1). Within each patient group, the decrease in systolic blood pressure was 8.2% (95% CI −20.3 to 1.0; *p* = 0.070) and 9.6% (95% CI −24.5 to −0.4; *p* = 0.045) for COPD (150.0 ± 13.2 vs. 140.4 ± 19.6 mmHg) and T2DM (151.7 ± 24.5 vs. 139.3 ± 15.9 mmHg), respectively. For diastolic blood pressure, no significant effect of the training intervention was observed (90.5 ± 14.1 vs. 87.5 ± 10.9 mmHg; ∆ = −3.0 mmHg, 95% CI −9.5 to 3.5; *p* = 0.078). Neither the COPD (98.9 ± 15.2 vs. 90.5 ± 9.7 mmHg; ∆ = −8.4 mmHg, 95% CI −18.6 to 1.9; *p* = 0.095) nor the T2DM (84.9 ± 10.6 vs. 85.5 ± 11.6 mmHg; ∆ = −0.6 mmHg, 95% CI −8.5 to 9.7; *p* = 0.890) group had any significant change (Figure 1).

### 3.2. Body Mass

No effect of the training intervention on body mass was observed in the total sample (n = 15), with no significant changes in either the obstructive pulmonary disease (COPD) group (n = 6) or the type 2 diabetes mellitus (T2DM) group (n = 9). Body mass in the total group changed from 94.4 ± 24.8 kg to 94.0 ± 23.9 kg (∆ = −0.4 kg, 95% CI −1.8 to 1.0; *p* = 0.518). Similarly, no significant change was observed in COPD (75.2 ± 16.9 vs. 75.7 ± 16.5 kg; ∆ = 0.5 kg, 95% CI −1.4 to 2.4; *p* = 0.519) or T2DM (107.3 ± 20.9 vs. 106.2 ± 20.4 kg; ∆ = −1.1 kg, 95% CI −3.2 to 1.1; *p* = 0.291).

### 3.3. Functional Capacity

**Figure 2:**
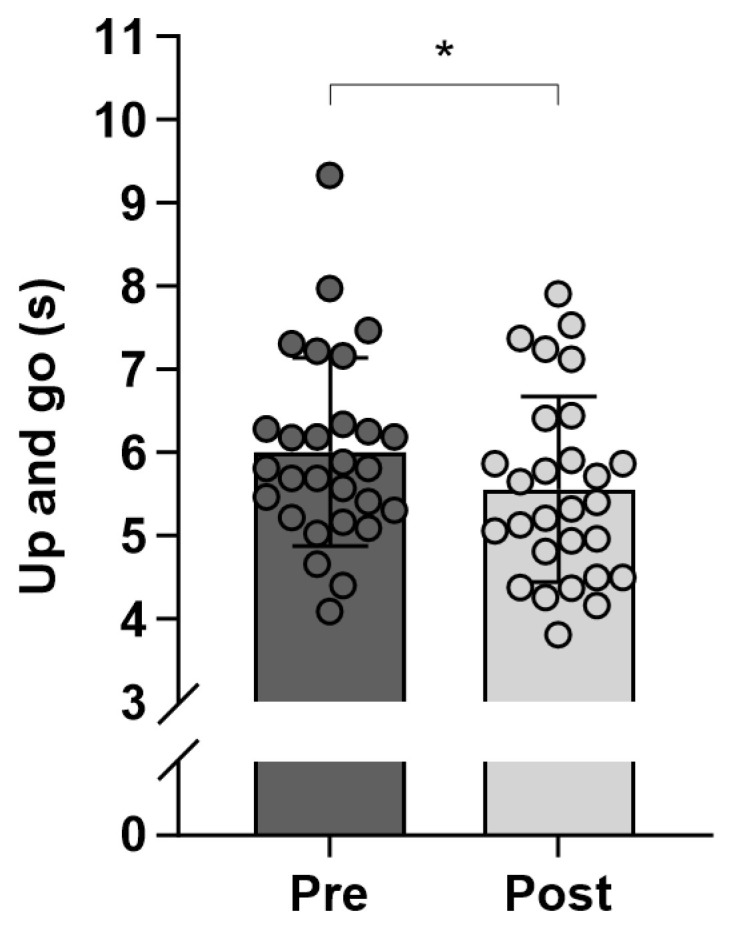
Time to complete the 2.45 m “Up and Go” test within a group of obstructive pulmonary disease (COPD) (n = 12) and diabetes mellitus type 2 (T2DM) (n = 16) patients before (Pre) and after (Post) 12 weeks of team sports and circuit training conducted in a municipal health center. Data are presented as individual values and mean ± SD. * Denotes difference (*p* < 0.05) between Pre and Post.

After the training intervention, time to complete the 2.45 m “Up and Go” test decreased by 7.5% (*p* < 0.001) compared to before the intervention (6.0 ± 1.1 vs. 5.6 ± 4.8 s; Figure 2). Within each patient group, the decrease in time to complete the 2.45 m “Up and Go” test was 7.3% (*p* = 0.011) and 7.5% (*p* = 0.005) for the COPD (6.3 ± 1.4 vs. 5.9 ± 1.3 s) and T2DM (5.7 ± 0.9 vs. 5.3 ± 0.9 s) groups, respectively.

After the training intervention, the distance covered in the 6 min walking test (6 MWT) was 3.0% longer (*p* = 0.082) compared to before the training intervention (538 ± 67 vs. 555 ± 74 m). Within the T2DM group, an increase (*p* = 0.04) of 3.6% was observed during the intervention (541 ± 76 vs. 560 ± 76 m), whereas a non-significant increase was found for the COPD group (532 ± 52 vs. 540 ± 96 m).

There were no changes during the intervention period for the 30 s chair stand, biceps curl, time to complete five sit-to-stand repetitions, and handgrip strength test (Table 3).

## 4. Discussion

The main findings in the present study were that the training intervention, mainly consisting of team sport, conducted in a municipality health center led to a marked decrease in systolic blood pressure in the hypertensive COPD and T2DM patients (10 and 12 mmHg, respectively). The study also showed positive effects on performance, as time to complete the 2.45 m “Up and Go” test was shorter and the distance covered during a six-minute walking test was longer after compared to before the intervention period (5.6 vs. 6.0 s and 555 vs. 539 m, respectively) for both patient groups.

The marked decrease in systolic blood pressure of 10 and 12 mmHg in the COPD and T2DM patient groups, respectively, during the 12-week intervention is similar to the finding (12 mmHg) by Andersen et al. [17] examining the effect of 3 months of regular soccer training conducted as small-sided games in thirty-one untrained males with mild-to-moderate hypertension. Furthermore, nine hypertensive postmenopausal women completed 10 weeks of biweekly small-sided floorball training and decreased systolic BP by 15 mmHg [18]. However, in these studies the subjects were not recruited through the practitioners, and none had COPD or T2DM, where the effect of exercise training on systolic blood pressure has been difficult to demonstrate in other studies. Thus, Gouzi et al. [19] did not find a decrease in systolic blood pressure in 49 COPD patients aged 62 years with an initial systolic blood pressure of 132 mmHg conducting 20 sessions of 60–90 min of moderate-intensity cycling over 4–6 weeks, and in a study by Li et al. [20] T2DM patients aged 38 years, with baseline systolic blood pressure being around 140 mmHg, did either high-intensity interval training or moderate-intensity continuous training for 12 weeks, and neither group had a decrease in systolic blood pressure. Furthermore, Dobrosielski et al. [21] investigated the effect of a 26-week program of combined aerobic and power training with sessions three times per week lasting 45–60 min on 51 participants with T2DM (33 men and 18 women) with an average age of 57 years and baseline systolic blood pressure of 127 mmHg, and they did not find any change in systolic blood pressure. In addition, Møller et al. [10] studied the effect of team sports in non-hypertensive patients in a similar environment as in the present study and did not find a change in systolic blood pressure. Nevertheless, Ambelu and Terifi [22] examined the impact of aerobic training, power training, and combined training on patients with T2DM aged 40–45 years with a baseline systolic blood pressure of around 155 mmHg (seven men and three women in each group) and found a decrease in systolic blood pressure in all three groups (17, 14, and 25 mmHg, respectively).

Taken together, it appears that team sports for hypertensive patients with T2DM and COPD can be carried out in a ’real-life setting’ and have a significant positive effect on blood pressure. The decrease in systolic blood pressure of 10–12 mm Hg is of high clinical relevance, as a decrease of 10 mmHg in systolic blood pressure is associated with ∼40% lower risk of stroke death and ∼30% lower risk of death from ischemic heart disease [23]. The cause of the reduced blood pressure remains speculative. A study by Sjúrðarson and colleagues reported cardiac remodeling, including a 10% increase in left ventricular mass index, in hypertensive premenopausal women following 15 weeks of soccer training [24]. They also observed enhanced filling of the left ventricle (reflected by an increase in the E/A ratio), which ensures more efficient cardiac output and could therefore contribute to the observed decrease in systolic blood pressure.

Both the T2DM and COPD groups had a non-significant decrease in diastolic blood pressure, which would suggest that while team sports effectively lower systolic blood pressure in hypertensive patients with T2DM and COPD, the impact on diastolic blood pressure may be less pronounced. Nevertheless, it appears that sustained engagement in team sports offers broader benefits, which are particularly valuable for patients with T2DM and COPD. Therefore, incorporating team sports into routine treatment plans could enhance both physical and social well-being, supporting comprehensive health management in these populations.

The time for the 2.45 m “Up and Go” test decreased by 6.7% (Figure 2), and the distance covered in the six-minute walk test increased by 3.0% during the 12-week intervention period (Table 3). Others have also found improved performance after a period of team sports with similar age groups. Thus, Duncan et al. [25] found that 12 weeks of recreational small-sided football training twice a week in elderly (66 years) men improved the 2.45 m “Up and Go” test and the six-minute walk test by 24% and 11%, respectively. Likewise, elderly (69 years) men playing floorball twice a week for 12 weeks improved in the six-minute walk test (4%) but did not have a change in the 2.45 m “Up and Go” test [11]. The lack of significant changes in muscle endurance tests (e.g., 30 s chair stand, biceps curl) may be due to the circuit training’s load and volume not aligning with standard resistance training periodization. It is recommended that for improved muscle endurance, the rep range is over 15–20 reps, but in this study, the participants only did around 10–15 reps for each set. Hereby the volume was also low for the circuit training during the 12 weeks (25% of the time) due to the predominance of team sports [26]. Furthermore, de Lima et al. [27] conducted a randomized controlled trial with COPD patients divided into three groups: (1) a functional training group combining resistance, aerobic, and functional exercises; (2) a conventional training group focusing on resistance and aerobic training; and (3) a usual care group receiving standard respiratory physiotherapy. Only in the functional training group did they find an increase (40 m) in the six-minute walk test. Similarly, Janssen and Connelly [28] reported in a systematic review that various exercise interventions improved six-minute walk performance by up to 60 m in adults with type 2 diabetes. No significant changes were observed in other measures of functional capacity in the present study. Apparently, team sports and circuit training led to improved performance of the COPD and diabetic patients in endurance capacity and ability to rise and walk fast, but not in specific muscle endurance.

While this study demonstrated significant benefits of the 12-week team sports intervention on systolic blood pressure and functional capacity, several limitations must be considered. Although the intervention primarily focused on team sports, circuit training was included, accounting for approximately 25% of the total training time. Thus, the reductions in systolic blood pressure cannot be exclusively attributed to team sports.

The absence of a control group in this study represents a limitation, as it cannot be definitively ruled out that other factors may have influenced the observed changes. However, it is unlikely that maintaining a normal lifestyle would result in the significant reductions in blood pressure observed in this study. Andersen and colleagues, for example, found no changes in systolic or diastolic blood pressure in a group receiving physician-guided traditional recommendations for cardiovascular risk factor modification, in contrast to a football training group that demonstrated marked improvements [17].

## 5. Conclusions

The 12-week intervention demonstrated that team sports, conducted in a municipal health center, effectively reduced systolic blood pressure and improved functional capacity (“Up and Go” test) in hypertensive COPD and T2DM patients. These findings suggest that team sports can serve as an alternative to traditional exercise therapies, combining physical, social, and psychological benefits. The study supports the integration of team sports into treatment plans for managing hypertension in patients with chronic conditions, highlighting their potential for long-term adherence and enjoyment.

## 6. Perspectives

This study was conducted in a municipal health center, with training sessions guided by health center personnel following instructions from the scientific staff at the Copenhagen Center of Team Sport and Health, University of Copenhagen, Denmark. Team sports were introduced as an alternative to traditional activities, such as cycling exercises. In interviews, participants expressed that team sports were enjoyable and naturally encouraged movement. One participant remarked, “*It has been fantastic... you really get to move*”, while another noted that the intensity of throwing the ball elevated her heart rate. The participants also emphasized the positive social dynamics that developed through their interactions during and after the games. Notably, after completing the 12-week program, participants chose to continue engaging in team sports together. This suggests that team sports is not only an effective means of increasing physical activity in patients with chronic obstructive pulmonary disease (COPD) and type 2 diabetes mellitus (T2DM) but also foster long-term adherence to physical activity through enjoyment and social engagement.

## 7. Practical Applications

Accessible facilities, appropriate equipment, and trained facilitators are necessary to deliver socially inclusive group activities that foster adherence through social engagement [29]. A study by Engdal et al. [30] highlighted the importance of volunteer training on inclusivity and health promotion. Scalability can be achieved using standardized templates, sustainable funding models, and targeted community awareness campaigns, facilitating widespread adoption and improved health outcomes [30].

## Figures and Tables

**Table 1:** Baseline characteristics of the COPD and T2DM patients.

	COPD (n = 12)	T2DM (n = 16)
Age (years)	69.4 ± 5.9	64.5 ± 10.7
Sex (Male n/Female n)	8/8	5/5
Body mass (kg)	75.2 ± 16.9	107.3 ± 21.0
Systolic Blood Pressure (mmHg)	150.0 ± 13.2	151.7 ± 24.5
Diastolic Blood Pressure (mmHg)	98.8 ± 15.2	84.9 ± 10.6

Characteristics of the patients in the group with obstructive pulmonary disease (COPD) and diabetes mellitus type 2 (T2DM). Data are presented as mean ± SD.

**Table 2:** Structure of the 12-week training intervention.

Week	Duration	Day 1 (Monday)	Day 2 (Wednesday)
**1**	60 min	Ball handling	Ball handling
**2**	60 min	Cone Ball	Cone Ball
**3**	60 min	Floorball	Circuit training
**4**	60 min	Floorball	Circuit training
**5**	60 min	Cone Ball	Floorball
**6**	60 min	Cone Ball	Floorball
**7**	60 min	Circuit training	Cone Ball
**8**	60 min	Circuit training	Cone Ball
**9**	60 min	Floorball	Cone Ball
**10**	60 min	Floorball	Cone Ball
**11**	60 min	Floorball	Circuit training
**12**	60 min	Floorball	Circuit training

**Table 3:** Effect of 12 weeks of team sports training and circuit training on functional tests within a group of obstructive pulmonary disease (COPD) and diabetes mellitus type 2 (T2MD) patients as well as both patient groups together (ALL).

	Chair Stand (s)	Five Rep (s)	Up and Go (s)	Bicep Curl (n)	Hand Grip (kg)	Six MWT (m)
**COPD**	n = 12	n = 12	n = 12	n = 12	n = 12	n = 2
**Pre**	14.9 ± 4.5	10.6 ± 3.1	6.3 ± 1.4	15.8 ± 3.8	32.7 ± 10.8	532.2 ± 51.5
**Post**	15.5 ± 5.2	10.1 ± 2.9	5.9 ± 1.3 *	17.0 ± 3.7 *	34.3 ± 12.3	539.8 ± 95.8
**∆ (95% CI)**	0.6 (−1.0 to 2.2)	−0.5 (−1.6 to 0.6)	−0.5 (−0.8 to −0.1)	1.2 (0.2 to 2.1)	1.7 (−0.5 to 3.8)	7.6 (−390.5 to 405.6)
***p*-value**	0.437	0.337	0.011	0.023	0.112	0.849
**T2DM**	n = 16	n = 16	n = 16	n = 16	n = 16	n = 8
**Pre**	14.6 ± 2.6	10.5 ± 1.9	5.8 ± 0.9	19.9 ± 5.4	33.1 ± 8.0	540.9 ± 75.5
**Post**	14.8 ± 2.7	10.2 ± 2.0	5.3 ± 0.9 **	20.6 ± 5.1	33.3 ± 9.9	560.2 ± 75.6 *
**∆ (95% CI)**	0.2 (−1.3 to 1.7)	−0.3 (−1.1 to 0.5)	−0.4 (−0.7 to −0.2)	0.8 (−2.0 to 3.5)	0.2 (−2.1 to 2.4)	19.3 (1.3 to 37.2)
***p*-value**	0.795	0.478	0.005	0.570	0.862	0.040
**All**	n = 28	n = 28	n = 28	n = 28	n = 28	n = 10
**Pre**	14.8 ± 3.5	10.5 ± 2.5	6.0 ± 1.1	18.1 ± 5.1	32.9 ± 9.1	538.7 ± 66.9
**Post**	15.1 ± 3.9	10.1 ± 2.4	5.6 ± 1.1 ***	19.1 ± 4.8	33.7 ± 10.8	555.1 ± 74.0
**∆ (95% CI)**	0.4 (−0.7 to 1.4)	−0.4 (−1.0 to 0.2)	−0.5 (−0.7 to −0.3)	1.0 (−0.6 to 2.5)	0.8 (−0.7 to 2.3)	16.3 (−2.7 to 35.4)
***p*-value**	0.483	0.223	<0.001	0.228	0.272	0.082

Data are presented as mean ± SD and changes (∆ (95% CI)). * Denotes difference at *p* < 0.05 between Pre and Post, ** denotes difference at *p* < 0.01 between Pre and Post, *** denotes difference at *p* < 0.001 between Pre and Post.

## Data Availability

The original contributions presented in this study are included in the article. Further inquiries can be directed to the corresponding author.
